# Estrogen-dependent effects of vagotomy and endogenous opioids on temporomandibular joint-responsive neurons in trigeminal subnucleus caudalis

**DOI:** 10.1016/j.jphyss.2025.100046

**Published:** 2025-10-17

**Authors:** Akimasa Tashiro, David A. Bereiter, Yuna Kani, Yuji Morimoto

**Affiliations:** a Department of Physiology, National Defense Medical College, Namiki 3-2, Tokorozawa City, Saitama 359-8513, Japan; b Department of Diagnostic and Biological Sciences, University of Minnesota School of Dentistry, 515 Delaware St. SE, Minneapolis, MN 55455, USA

**Keywords:** Temporomandibular joint, Trigeminal subnucleus caudalis, Vagus nerve, Estrogen, Endogenous opioids

## Abstract

Temporomandibular joint (TMJ) disorders are common orofacial pain conditions influenced by multiple factors. This study examined how vagotomy and endogenous opioids affect TMJ-responsive neurons in the trigeminal subnucleus caudalis/cervical junction (Vc/C_1–2_) in female rats under different estrogen levels. Under low estrogen, cervical vagotomy enhanced TMJ unit responses to levels seen in high estrogen conditions but had no additional effect under high estrogen. Vagotomy did not change chemical stimulation (ATP) thresholds or spontaneous activity, suggesting a central neural mechanism. Responses to mechanical stimulation of the skin over the TMJ were unaffected. Naloxone increased ATP-evoked responses under low estrogen but had no added effect after vagotomy or under high estrogen. Naloxone also did not alter spontaneous activity or mechanical responses. These findings indicate that vagal input and endogenous opioids significantly modulate TMJ neuron activity under low estrogen, while high estrogen levels limit further excitation, implying estrogen-dependent regulation of vagus and opioid effects on Vc/C_1–2_ neurons.

## Highlights

• Vagotomy enhanced TMJ neuron responses under low but not high estrogen.
• Naloxone increased ATP-evoked TMJ responses only under low estrogen.
• Vagotomy and naloxone effects suggest shared opioid-related pathways.
• Estrogen status regulates vagal and opioid modulation of TMJ units.
• Hormonal influence is critical in vagus- and opioid-mediated pain control.

## Introduction

The junction region between trigeminal subnucleus caudalis and the upper cervical cord (Vc/C_1–2_) shares many properties with the spinal dorsal and is closely associated with pain processing [1], [2]. Temporomandibular disorders (TMD) are a diverse family of conditions marked by pain in the temporomandibular joint (TMJ) and adjacent masticatory muscles [3], [4] and pain is the main reason to seek medical attention for TMD [5]. Sensory nerves that supply the TMJ and jaw muscles terminate on Vc/C_1–2_ neurons [6], [7] that also receive convergent input from surrounding tissues [8], [9], [10]. Sensory branches of the vagus nerve (Xn) also project to the Vc/C_1–2_ region [11], [12]. TMJ units at Vc/C_1–2_ are activated by convergent input by Xn stimulation [10], while TMD patients often report jaw pain referred from tissues supplied by the vagus nerve [13], [14]. Stimulation of Xn is well established as a non-invasive method to modify pain sensation [15], [16]. Similarly, Xn stimulation reduces release of glutamate [17] and neural activity in the Vc/C_1–2_ region [18] in animal models of craniofacial pain.

The mechanisms underlying Xn-mediated pain modification likely involve multiple pathways [19]. One possible mechanism involves Xn activation and the release of endogenous opioids. Endogenous opioids consist of three families of peptides: β-endorphine, enkephalins and dynorphins [20]. Endomorphin-2 is an endogenous opioid peptide with a high affinity for the mu-opioid receptor and is expressed by a significant number of TG [21] and nodose ganglion neurons [22]. Axon terminals for endomorphin-2[23 and for enkephalins [24] are densely distributed at the Vc/C_1–2_ region. Opioid receptors also are moderately to densely expressed at the Vc/C_1–2_ region [25] and in trigeminal (TG) and nodose ganglion neurons [26]. A prominent feature of TMD is the higher prevalence in women than men [3], [27]. While in animal studies, the inhibitory effect of morphine on stimulus-evoked TMJ unit responses at Vc/C_1–2_ [28] and TG responses to jaw movement [29] were dependent on estrogen status in female rats. Estrogen receptors (ER) are densely expressed on neurons at the Vc/C_1–2_ region [30], [31] and 40–60 % of ER-positive neurons colocalized preproenkephalin mRNA [32]. The goal of the present study was to determine if estrogen status played a significant role in modifying TMJ unit activity following cervical vagotomy or after inhibition of opioid receptors by naloxone.

Previously, we reported that exogenous opioids act outside the Vc/C_1–2_ region to inhibit ATP-induced excitation of TMJ-responsive neurons in an estrogen-dependent manner [28]. The basis for this effect is not known. The present study tests the hypothesis that vagus nerve input is necessary to recruit endogenous opioid pathways, and that these pathways dependent on estrogen status.

## Methods

### Ethical approval

The study protocols were authorized by both the Committee of Research Facilities for Laboratory Animal Science at the National Defense Medical College (Japan) and the Institutional Animal Care. The protocols followed the guidelines established by The National Institutes of Health guide for the care and use of laboratory animals (PHS Law 99–158; revised, 2002).

### General and endocrine procedures

Adult age-matched ovariectomized (OvX) female Sprague-Dawley rats weighing 250–320 g (SLC in Shizuoka, Japan, n = 55) were used within 14 days after surgery. Rats were given daily injections of either low-dose (LE, 2 µg, s.c.) or high-dose (HE, 20 µg, s.c.) 17α-estradiol-3-benzoate (E2; Sigma, St. Louis, MO) dissolved in 200 µl sesame oil for 2 days. The LE and HE replacement regimens were chosen to mimic E2 plasma levels during diestrus and proestrus, respectively [33]. The estrogen status was assessed on the experiment day from vaginal smear cytology obtained through gentle lavage. LE rat samples had more than 80 % small nucleated leukocytes and consistent with low-estrogen status, whereas samples from HE rats displayed mainly large nucleated epithelial cells and consistent with high-estrogen status [34]. Previously we reported that this dose regimen resulted in plasma E2 levels of less than 20 and 50–100 pg/ml, for LE and HE rats, respectively [35].

### Animal preparation: electrophysiology

The rats were anesthetized with isoflurane (3.0 %–5.0 %). Catheters were inserted into the right femoral artery for blood pressure monitoring, the femoral vein for naloxone administration, and the right jugular vein for administration a paralytic agent at the time of recording. The cervical vagal nerve trunks were cut bilaterally, distal to the emergence of the superior laryngeal nerve (vagotomy; VgX). Following tracheotomy, animals were ventilated with oxygen-enriched room air (>80 % O₂) and maintained under isoflurane (1.0 %–1.5 %). Rats were infused with gallamine triethiodide, a short-acting paralytic agent (25 mg/kg/h), only during the neural recording session. Expiratory end-tidal CO_2_ (3.5 %–4.5 %), mean arterial pressure (90–120 mm Hg), and body temperature (38°C) were monitored and kept within normal limits.

The animals were positioned in a stereotaxic frame and the dorsal portion of the C1 and C2 vertebrae was removed to expose the upper cervical dorsal horn. The brainstem surface was kept moist with warm mineral oil. The left temporalis muscle was reflected, revealing the lateral pterygoid muscle and connective tissue on the dorsal aspect of the posterior mandibular condyle. The caudal portions of the trigeminal subnucleus caudalis (Vc) and upper cervical (C1–2) spinal cord, 4–7 mm caudal to the obex, were explored ipsilateral to the exposed condyle for TMJ-responsive units, with the entrance of the C2 rootlet serving as a landmark for electrode placement. Single-unit extracellular recordings were made with tungsten microelectrodes (5 MΩ; Frederick Haer Inc., Bowdoinham, ME, USA). Unit activity was amplified, discriminated (model WD-2, Bio Research Center Co., Nagoya, Japan), saved, and analyzed offline with a PowerLab interface and LabChart software (ADInstruments, Bella Vista, Australia).

All units included for further analyses responded vigorously to gentle mechanical probing of the exposed dorsal surface of the condyle and surrounding muscles (“TMJ units”). All TMJ units were categorized as nociceptive specific and were not activated by brush of the skin overlying the TMJ but were excited by a “press” (arterial clip, approximately 20 mm²) and “pinch” stimulus (shorter and stiffer arterial clip, approximately 15 mm²) applied to the facial skin. The press stimulus caused mild pain, whereas the pinch stimulus was clearly painful when applied to the investigator’s forearm skin. The average response magnitudes to press and pinch were determined over a 10 s stimulus period.

### Experimental design

Thirty-two TMJ units, from 32 rats, were recorded from the superficial laminae (<200 µm from the dorsal surface), 1.5 mm rostral to the level of the C2 rootlet entrance and recording sites were confirmed by histological examination. The loss of corneal and hindlimb withdrawal reflexes confirmed an adequate depth of anesthesia. A single TMJ unit was recorded in each animal preparation. The face and neck were explored for convergent cutaneous input. The high-threshold cutaneous receptive field (RF) area of each TMJ unit was mapped using a small blunt forceps (approximately 3 mm²) and transferred onto standardized rat face drawings for quantification by ImageJ software. Following cutaneous RF stimulation and mapping, a guide cannula (26 gauge) was inserted into the TMJ joint space (∼3 mm deep) via a dorsal approach and directed toward the posterior aspect of the mandibular condyle to allow for delivery of chemical stimuli. Test solutions were injected through a microsyringe connected by polyethylene tubing to an inner cannula (33 gauge) that protruded approximately 0.5 mm from the end of the guide cannula.

In the first series the effects of estrogen status (LE, HE) and vagotomy (VgX) were assessed on the responses to cutaneous and intra-TMJ stimuli. Intra-TMJ stimulation consisted of phosphate-buffered saline (PBS, pH 7.4) followed by increasing concentrations of adenosine triphosphate (ATP, 0.01–1 mM, 20 µl) administered slowly over 30 s at 20-min intervals. This range of ATP concentrations evoked cutaneous [36] and muscle [37] pain sensation in humans. ATP selectively excites small diameter sensory neurons from deep tissues [38], [39] and can be repeatedly applied as a test stimulus without causing tachyphyaxis [28]. In a second series, the role of endogenous opioids, estrogen status and VgX on cutaneous and intra-TMJ evoked responses was assessed by administration of effects of the opioid receptor antagonist naloxone-HCl (0.1, 1 mg/kg, iv) 10 min prior to stimulation in a separate group of 23 rats. Pinch was used to assess responses to cutaneous stimulation, while repeated intra-TMJ injections ATP (1 mM) were delivered at 20 min intervals was used to assess responses to TMJ input.

### Data analysis

The estrogen status of the rat was not known to the investigator prior to the experiment. LabChart was used to record and display neural data as peristimulus time histograms (PSTH) of spikes per second (1-s bins) and then exported to a spreadsheet and analyzed offline. Spontaneous activity (SA, spikes/s) was calculated as the average spike count during a 1-min period preceding each ATP stimulus [40]. Naloxone effects on SA were determined from the average spike count over 1 min before and after drug administration. ATP-evoked responses were determined as a total response magnitude (Rmag), which was calculated by subtracting the mean plus two times the standard deviation of background activity from the total spike count for each bin. The total Rmag for a given stimulus was calculated as the sum of spikes in contiguous bins where the spike count minus the background was positive and should be thought of as the “area under the curve” for each stimulus period. Response duration was defined as the time from stimulus onset to observe three consecutive bins with a positive spike count above the background and until the spike count fell again to background levels. Response latency was defined as the earliest time after stimulus onset to observe three consecutive 1-s bins that exceeded background activity. Units that did not show three consecutive bins with positive Rmag values within 100 s of stimulus onset were considered unresponsive to that stimulus. All units in this study were defined as ATP-responsive and displayed a total Rmag that exceeded the response to PBS by more than 50 %. Two-way ANOVA with repeated measures on 1 factor (ATP concentration) was used to assess the total Rmag, duration, and latency responses to increasing concentrations of ATP. Similarly, 2-way ANOVA assessed the responses to cutaneous stimuli (i.e., brush, press and pinch) and quantified as spikes per 10 s. Chi-square analyses determined if the ATP threshold concentration was different for LE and HE groups with versus without VgX. One-way ANOVA determined the effects of VgX on SA and on the cutaneous high-threshold RF area. Statistical comparisons were made using GraphpadPrism (v. 10.4) and individual comparisons were made after ANOVA by Tukey’s or the Newman–Keuls test. Quantitative data are shown as mean±SEM. A p-value of < 0.05 was considered statistically significant.

### Histology

**Fig. 1:**
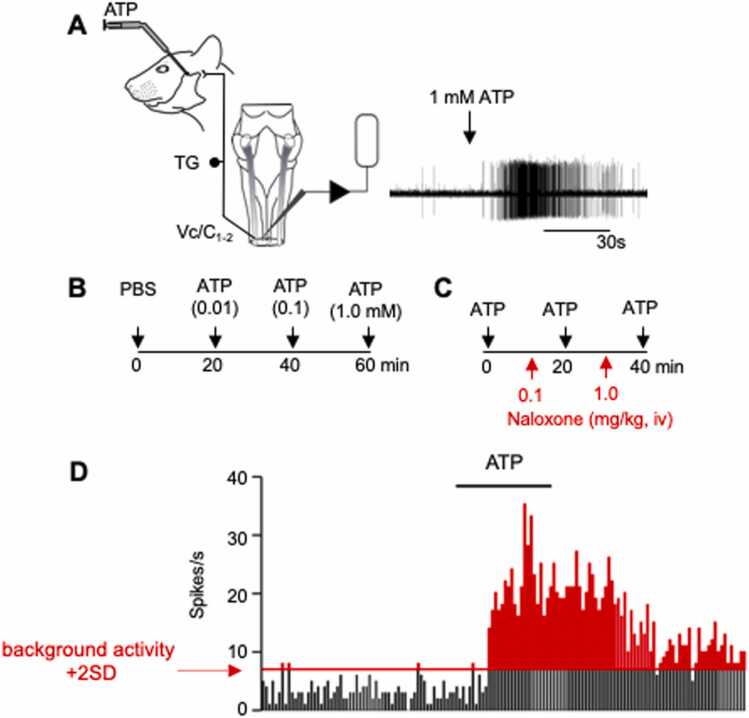
(A) General setup for neuronal recording at the Vc/C_1–2_ region with intra-TMJ test injections of ATP. (B) Design for intra-TMJ injections of ATP at 20 min. intervals. ATP concentrations consisted of (0.01, 0.1, 1 mM, 20 µl) applied as a cumulative dose regimen. (C) Design for assessing the role of endogenous opioids on ATP-evoked neuronal responses. Test stimuli consisted of repeated intra-TMJ injections of ATP (1 mM) at 20 min intervals. Naloxone (0.1–1 mg/kg i.v.) was administered 10 min prior to the ATP test stimulus. (D) Calculation of response magnitude (Rmag). Background activity was defined as the mean spike rate + 2 SD (dotted line). The red area indicates Rmag, obtained as the summed spike counts above background in contiguous bins (area under the curve). Response duration was defined as the time from stimulus onset until activity returned to background levels for three consecutive bins.

The recording location was marked electrolytically (5 µA, 20 s). The rats were deeply anesthetized with a bolus dose of pentobarbital sodium (60 mg/kg, i.v.), perfused the heart with saline followed by 10 % formalin and prepared histologically to recover the sites. The recovered lesion sites were aligned with a standard series of rat brainstem outlines. Fig. 1

## Results

### Recording sites

**Fig. 2:**
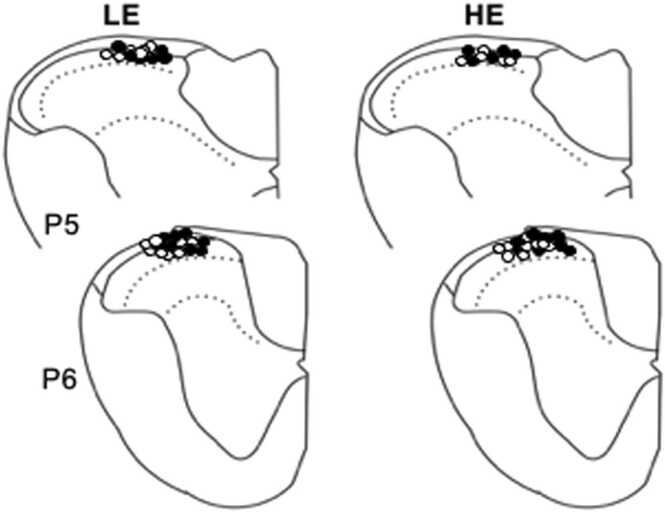
Recording sites of TMJ units from LE and HE rats in the superficial laminae at the Vc/C_1–2_ region. P5 and P6 refer to the approximate distance, in mm, from the obex. Sham (n = 28), filled circles = VgX (n = 27).

A total of 55 TMJ units were classified as nociceptive specific neurons and were included in one of two experimental series. All neurons were recorded in the superficial laminae of the Vc/C_1–2_ in LE and HE rats within 200 µm of the dorsal brainstem surface (Fig. 2).

*Series 1. ATP dose-effect responses*

### Effects of estrogen status and vagotomy (VgX) on ongoing unit activity

The ongoing activity of TMJ units prior to exogenous stimulation was similar across treatment groups (F_3,28_=0.94, p > 0.1. The mean spontaneous activity (SA) for neurons in LE groups was: Sham = 1.72 ± 0.55 spikes/s, n = 8; VgX = 2.00 ± 0.57 spikes/s, n = 8, p > 0.05) and in HE groups was: Sham = 3.31 ± 0.72 spikes/s, n = 8; VgX = 2.57 ± 0.98 spikes/s, n = 8.

### Effects of VgX on ATP-evoked unit responses

**Fig. 3:**
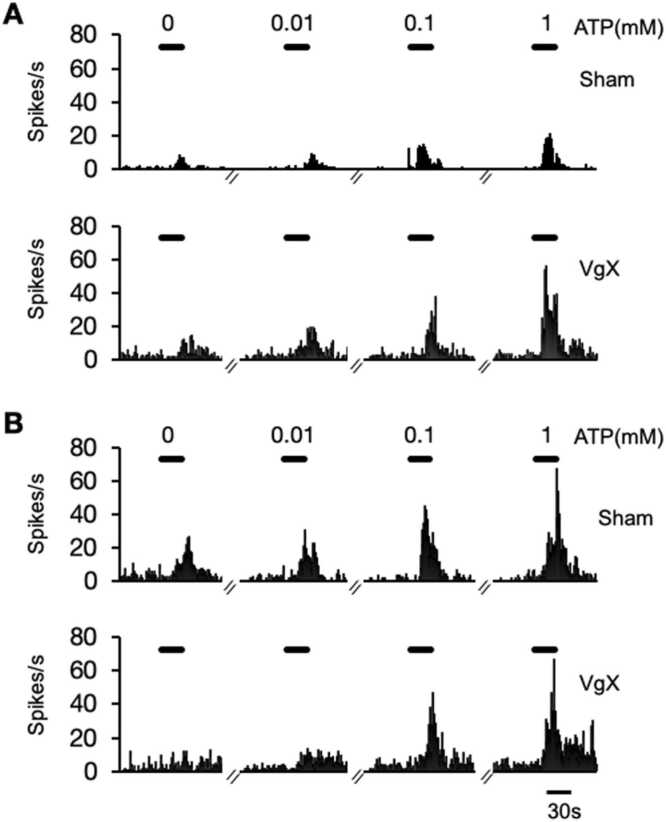
Peristimulus time histogram examples of the response to intra-TMJ injections of ATP in sham and VgX from LE (A), HE (B) female rats.

**Fig. 4:**
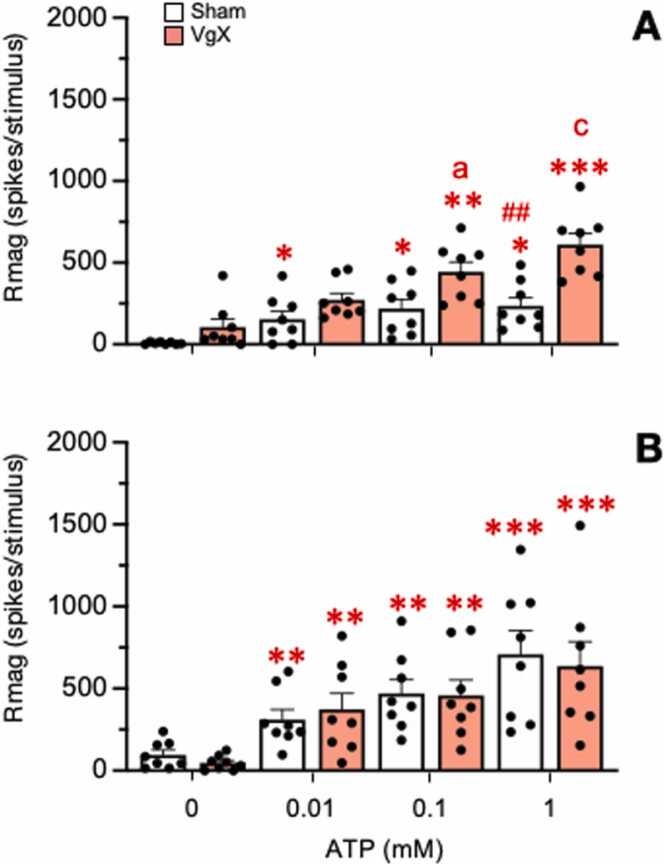
Total Rmag responses of Vc/C_1–2_ neurons to intra-TMJ injections of ATP in sham and VgX from LE (A) and HE (B) female rats. ATP (0.01, 0.1, and 1 mM) was injected at 20 min intervals. Sample sizes: LE Sham = 8; VgX = 8, HE Sham = 8; VgX = 8. Symbols and abbreviations: *p < 0.05, **p < 0.01, ***p < 0.001 versus vehicle injection (0 mM ATP); a = p < 0.05, c = p < 0.001 versus sham group; #p < 0.01 versus HE groups.

The responses to intra-TMJ injections of ATP (0.01, 0.1 and 1 mM) were recorded from a total 32 TMJ units of LE rats (Sham = 8, VgX = 8) and HE rats (Sham = 8, VgX = 8). The examples shown in Fig. 3 demonstrate dose-dependent ATP-evoked increases in firing frequency for LE (Fig. 3A) and HE rats (Fig. 3B) under Sham and VgX conditions. The results for total Rmag are summarized in Fig. 4 in which the treatment (F_3,28_=7.23, p < 0.001) and response (F_3,84_=69.37, p < 0.001) main effects were highly significant. Intra-TMJ injections of ATP as low as 0.1 mM caused significant increases in Rmag in all units (>100 % of baseline), independent of treatment. Individual comparisons revealed that VgX caused a further increase in ATP-evoked responses in LE rats (p < 0.025, Fig. 4A), while VgX did not cause further increases in ATP-evoked responses in HE rats (Fig. 4B). Comparison of responses of LE versus HE groups also were significant for sham animals at 1.0 mM ATP (p < 0.01), while the ATP-evoked Rmag values were similar for LE and HE rats after VgX. The threshold concentration of ATP needed to evoke a significant increase in Rmag was similar across all treatment groups (X^2^_df1_= 0.24, p > 0.66).

**Fig. 5:**
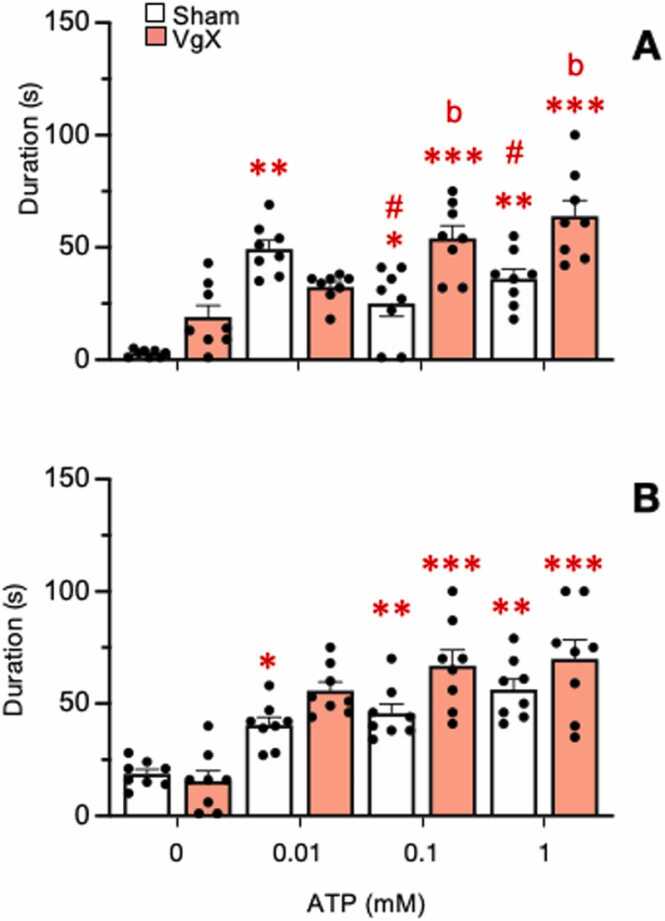
Response duration to ATP by Vc/C_1–2_ neurons in sham and VgX from LE (A) and HE (B) female rats. ATP (0.01, 0.1, and 1 mM) was injected at 20 min intervals. Sample sizes: LE Sham = 8; VgX = 8, HE Sham = 8; VgX = 8. Symbols and abbreviations: *p < 0.05, **p < 0.01, ***p < 0.001 versus vehicle injection (0 mM ATP); a = p < 0.05, b = p < 0.01, c = p < 0.001 versus sham group.

All groups also displayed a significant increase in response duration (Fig. 5, F_3,84_=80.24, p < 0.001). Treatment main effects also were significantly different (F_3,28_=12.48, p < 0.001). Individual comparisons indicated that response durations for LE rats further increased after VgX (p < 0.01, Fig. 5A), while VgX did not affect response duration in HE rats compared to sham animals (Fig. 5B). The ATP-evoked latency for each treatment group averaged 55.5 ± 6.4 s to 0.01 mM ATP and decreased to 9.5 ± 6.4 s to 1.0 mM ATP (F_3,84_=20.49, n = 32, p < 0.001) and was similar across all groups (F_3,28_=2.39, p < 0.1).

### Estrogen status and VgX on unit responses to mechanical stimuli

**Fig. 6:**
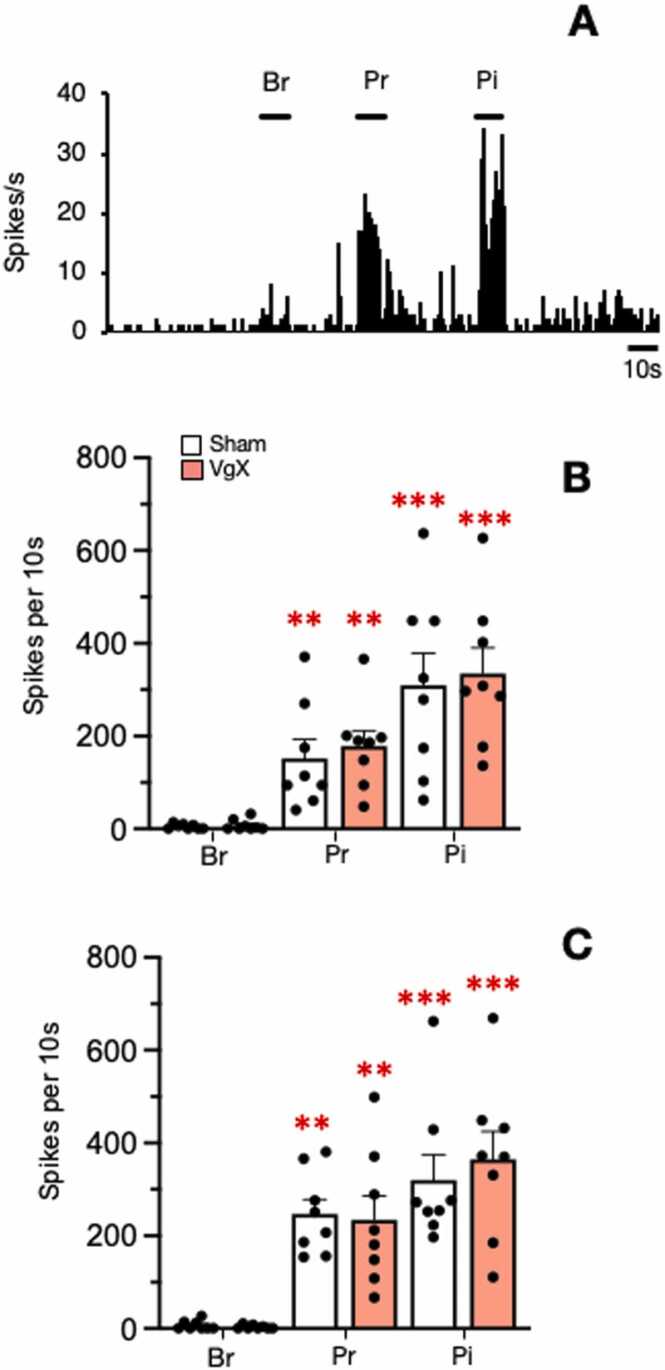
Peristimulus time histogram examples of the response to mechanical stimulation of the skin overlying the TMJ (A). Firing rate response to mechanical by Vc/C_1–2_ neurons in sham and VgX from LE (B) and HE (C) female rats. Stimuli were applied to skin overlying the TMJ for 10 s. Successive stimuli were applied at 10 min intervals. Sample sizes: LE Sham = 8; VgX = 8, HE Sham = 8; VgX = 8. Symbols and abbreviations: **p < 0.01, ***p < 0.001 versus brush; Br = brush,Pr = press, Pi = pinch.

All TMJ units responded to mechanical stimulation of the skin overlying the TMJ. Responses to brush, press and pinch caused a progressive increase in firing rate to a 10 s stimulus (Fig. 6). Two-way ANOVA revealed a marked increase in firing rate across all treatment groups (F_3,84_=20.49, n = 32, p < 0.001); however, treatment main effects were not significant (F_3,28_=0.46, p > 0.1). The high threshold convergent cutaneous RF area was mapped using a small forceps and averaged 0.89 ± 0.6 cm^2^ (n = 32, mean ± SD) and was not different across treatment groups (F_3,28_=031, p > 0.1). The RF typically extended anteriorly and ventrally from the TMJ and included areas supplied by the maxillary and mandibular branches of the trigeminal nerve.

### Series 2. Effects of naloxone (Nx) and VgX on ATP-evoked responses

**Fig. 7:**
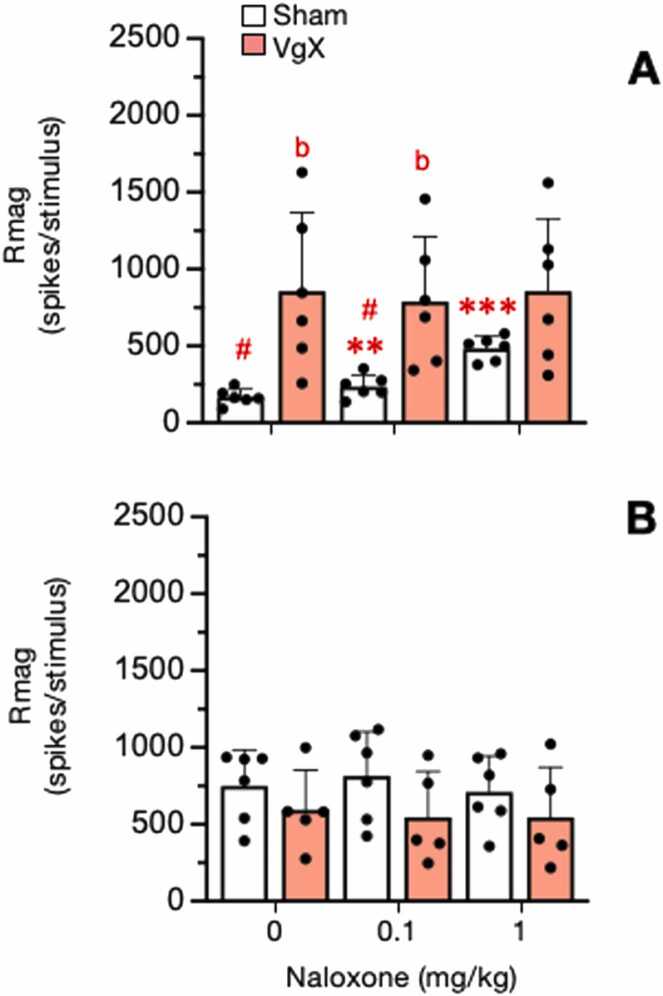
Effect of naloxone on ATP-evoked Rmag of Vc/C_1–2_ neurons in sham and VgX from LE (A) and HE (B) female rats. Intra-TMJ injections of 1 mM ATP served as the test stimulus. Naloxone (0.1 and 1 mg/kg, iv) was injected 10 min prior to the test stimulus. Sample size LE Sham = 6; VgX = 6, HE Sham = 6; VgX = 5. Symbols and abbreviations: **p < 0.01, ***p < 0.001 versus vehicle injection; b = p < 0.01 versus sham group; #p < 0.01 versus HE groups.

**Fig. 8:**
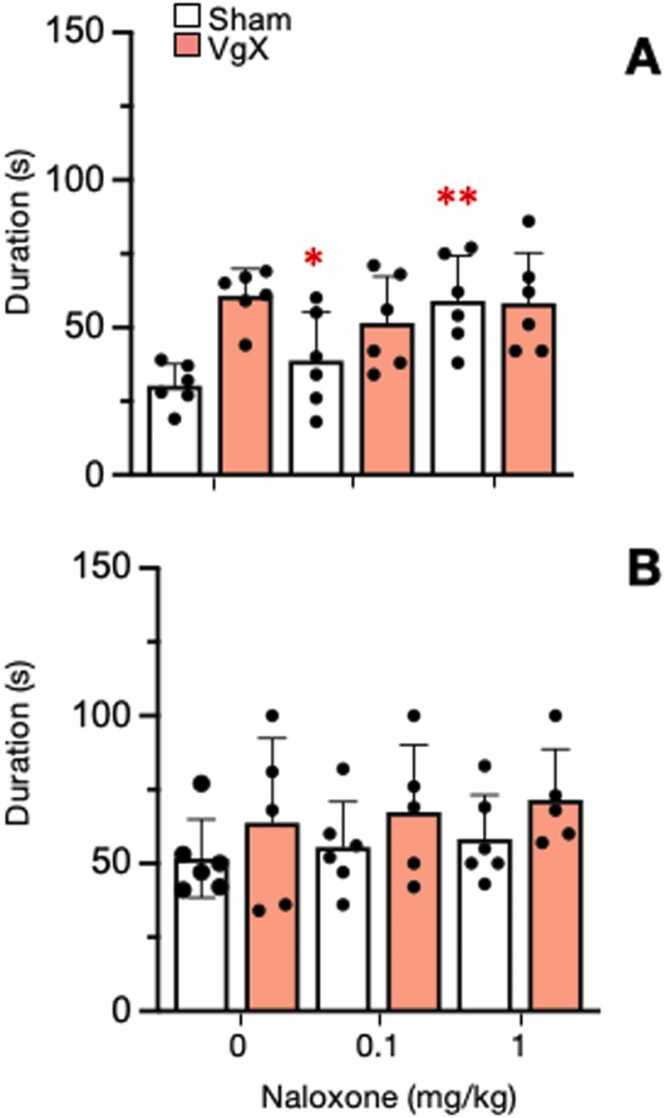
Effect of naloxone on ATP-evoked response durations of Vc/C_1–2_ neurons in sham and VgX from LE (A) and HE (B) female rats. Intra-TMJ injections of 1 mM ATP served as the test stimulus. Naloxone (0.1 and 1 mg/kg, iv) was injected 10 min prior to the test stimulus. Sample size LE Sham = 6; VgX = 6, HE Sham = 6; VgX = 5. Symbols and abbreviations: *p < 0.05, **p < 0.01 versus vehicle injection.

The role of endogenous opioids and VgX on ATP-evoked responses was assessed in separate groups of sham (n = 12) and VgX (n = 11) rats. Naloxone (0.1 and 1 mg/kg, iv) was injected 10 min prior to each 1 mM ATP test stimulus in LE and HE rats. The overall 2-way ANOVA revealed significant response (F_2,38_=12.75, p < 0.001) and treatment main effects (F_3,19_= 6.91, p < 0.001). Individual comparisons indicated that sham LE rats displayed a marked naloxone dose-dependent increase in ATP-evoked Rmag (F_2,38_=65.8, p < 0.001, while the elevated response in VgX LE rats was unchanged after naloxone (Fig. 7A). The ATP-evoked responses in HE rats (Fig. 7B) were similar after naloxone for both sham and VgX animals (F_2,38_ <1.5). Response duration also increased after naloxone in sham LE rats but not in VgX LE rats (Fig. 8A, F_2,38_=29.9, p < 0.001). Naloxone did not affect response duration in sham or VgX HE rats (Fig. 8B, p > 0.1). Naloxone did not significantly affect the ATP-evoked response latency in any treatment group (p > 0.1) and did not alter ongoing neural activity just prior to the ATP test stimulus (p > 0.1).

### Naloxone and mechanical stimulation

Pinch of the skin overlying the TMJ was applied before and after 1 mg/kg naloxone. The mean firing rate for a 10 s pinch was 187 ± 119 spikes/s and 284 ± 86 spikes/s (mean±sd) before and after naloxone, respectively (F_1,19_=1.35, p > 0.1) and were not different across all treatment groups (p > 0.1). Similarly, the RF area across all treatment groups averaged 0.81 ± 0.64 and 0.78 ± 0.6 cm^2^ (mean±sd) before and after 1 mg/kg naloxone, respectively (F_1,19_=0.13, p > 0.1). Individual comparisons for each treatment group revealed no intergroup differences (p > 0.1).

## Discussion

**Fig. 9:**
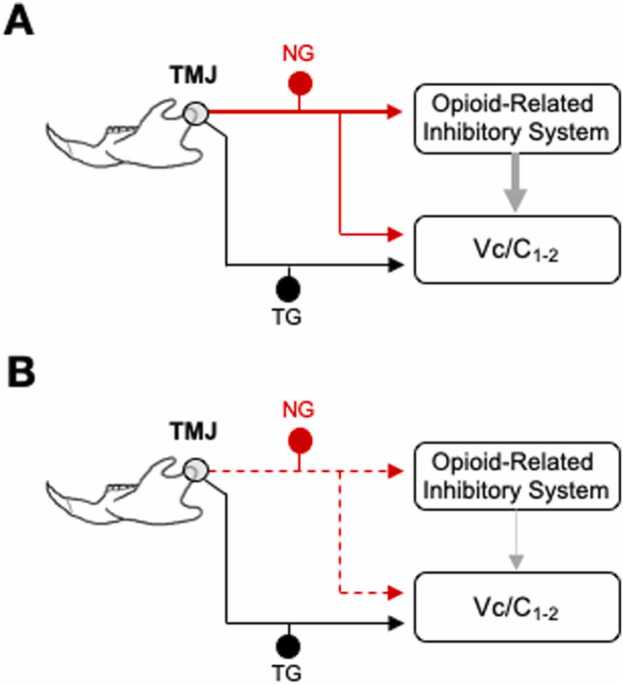
Proposed mechanisms of estrogen-dependent modulation of vagus nerve and opioid interactions. (A) Low-estrogen (LE) condition. Red arrows indicate vagus nerve afferents (NG, nodose ganglion), black arrows indicate trigeminal afferents (TG, trigeminal ganglion), and gray arrows indicate inhibitory pathways reducing ATP-evoked responses in TMJ-responsive Vc/C_1–2_ neurons. (B) High-estrogen (HE) condition. Both the vagus nerve input (red arrows) and the inhibitory effect (gray arrows) are weakened, as indicated by dotted lines. TMJ, temporomandibular joint; TG, trigeminal ganglion; NG, nodose ganglion.

This study revealed that estrogen status significantly influenced TMJ-evoked responses of Vc/C_1–2_ neurons following cervical vagotomy (VgX) and after naloxone inhibition of opioid receptors. Under low-estrogen (LE) conditions, VgX enhanced TMJ-evoked responses, while under high-estrogen (HE) conditions VgX had no significant effect on neuronal responses evoked by intra-TMJ injections of ATP. Similarly, naloxone caused a significant increase in ATP-evoked TMJ unit responses in sham LE rats that rose to the level of the responses seen by units in VgX LE rats. It was notable that the increase in ATP-evoked responses in LE sham rats after VgX were not further increased after naloxone, suggesting that vagal input and endogenous opioids act through common estrogen-dependent pathways. In contrast, the failure of VgX and naloxone to differentially affect responses in HE rats suggest that estrogen status is critical for modulation of intra-TMJ evoked responses by these pathways (Fig. 9). Since vagotomy did not alter ATP thresholds or spontaneous activity, strongly suggested that estrogen-dependent vagus nerve–opioid interactions are mediated by central mechanisms rather than by changes in peripheral excitability.

Vagal sensory fibers arise mainly from the nodose ganglion [55] and account for approximately 80 % of the vagus nerve [41]. Vagal afferents terminate centrally in the Vc/C_1–2_ and the paratrigeminal region (Pa5) [11], [12], where they converge with trigeminal inputs onto trigeminal brainstem neurons and thus provide a plausible basis for modulation of TMJ-evoked responses observed in this study.

### Vagus nerve and orofacial pain

The vagus nerve (Xn) is the largest viscerosensory nerve in mammals and consists of up to 80 % afferent fibers that terminate in several nuclear regions of the caudal brainstem [41]. In the trigeminal system, Xn afferent fibers terminate at the Vc/C_1–2_ and the paratrigeminal region (Pa5) [11], [12]. Clinical studies reveal that visceral pain that is transmitted by Xn afferent fibers often results in pain referred to the jaw region [13]. Indeed, acute jaw pain has been recognized by the American Heart Association as a warning sign for a heart attack [42]. Conversely, Xn stimulation has been used to successfully manage headache pain [43] and to reduce the pain of trigeminal neuralgia patients [44]. However, in control subjects low intensity Xn stimulation lowered heat pain thresholds after forearm application in control subjects [45]. In animal studies electrical [46] or noxious chemical stimulation of Xn afferents both excited and inhibited subsets of thalamic-projecting Vc/C_1–2_ neurons in the monkey [47]and the rat [48]. These studies suggest that the role of the Xn and orofacial pain is likely complex and is modified by multiple factors.

One likely modulatory factor is estrogen status since VgX effects on ATP-evoked responses by TMJ units were significantly increased only in LE rats, but not in HE rats. Previously we reported that response magnitude of TMJ-evoked responses by Vc/C_1–2_ neurons varied over the estrous cycle [49] and was enhanced by exogenous estradiol treatment [50]. Both estrogen and inflammation increased the excitability of TMJ-responsive TG neurons [51], while masseter muscle TG afferents displayed greater responses in female than male rats [52]. The site of action for estrogens to modify Xn-related TMJ unit activity is not certain, although it is likely to involve peripheral as well as sites within the brain. Estrogen receptors (ER) are found on trigeminal [34], [53], [54] and nodose ganglion neurons [55], while ER-positive neurons are found predominatly in superficial laminae [30], [31]. Previously we reported that estrogen treatment increases NMDA receptor and mGluR1-mediated neural activity the Vc/C_1–2_ region [35], [56] and inhibits GABA release [57] suggesting a local site of action. However, ER-positive neurons also are densely distributed in the periaqueductal gray area (PAG), a key region underlying descending control of nociceptive processing [58]. In contrast to ATP-evoked responses from the TMJ, vagotomy and naloxone did not significantly affect responses to noxious mechanical stimulation of the overlying cutaneous tissue. This is consistent with evidence that the endogenous opioid system exerts little influence on cutaneous nociceptive processing, whereas it plays a more prominent role in deep tissue pain, such as that originating from the TMJ. Previous work demonstrated that this inhibitory effect on deep tissue–evoked activity is estrogen-dependent [28]. The present findings further suggest that such estrogen-dependent endogenous opioid modulation of intra-TMJ nociception is mediated, at least in part, through vagal afferent input.

### Vagus nerve, endogenous opioid receptors and orofacial pain

Systemic administration of naloxone was used to assess the role of endogenously produced opioid peptides and estrogen status on TMJ-evoked Vc/C_1–2_ neuronal activity. Naloxone is non-specific antagonist that binds with moderate to high affinity to each of the three main opioid receptor subtypes [59]. Of the several known endogenous opioid peptides [20] that could play a role in the present study two peptides have particular close ties to the Vc/C_1–2_ region. Endomorphin-2 is a novel mu opioid receptor agonist that is produced by TG and nodose ganglion neurons [21], [22]. Immunopositive fibers for endomorphin-2 are densely distributed in the superficial laminae at Vc/C_1–2_ [23], [60]. Stimulation of Xn inhibited dura-evoked neurons at the Vc/C_1–2_ region, which was prevented by naloxone and mimicked by systemic injection of selective opioid receptor agonists [61]. Secondly, enkephalinergic neurons are densely distributed in superficial laminae at Vc/C_1–2_[24]. Immunohistochemical analyses revealed 60–70 % of Vc/C_1–2_ neurons that expressed preproenkephalin mRNA also expressed ER-like immunoreactivity [32]. Furthermore, acute injections of estradiol caused a marked increase in enkephalin mRNA in spinal dorsal horn [30]. Since a significant percentage of enkephalinergic neurons at Vc/C_1–2_ also express glutamic acid decarboxylase [24], the biosynthetic enzyme for gamma aminobutyric acid (GABA), and would suggest a local inhibitory effect of estrogen on nociceptive processing. Indeed, local application of estradiol to the dorsal surface of Vc/C_1–2_ caused a dose-dependent reduction in ATP-evoked TMJ unit activity [62]. These findings may seem contradictory; however, they highlight the complex nature of estradiol modulation on Vc/C_1–2_ neurons. Depending on receptor distribution, neuronal subtype, and experimental conditions, estradiol can exert both facilitatory and inhibitory influences, indicating that its effects are multifaceted rather than uniform [30], [31], [62]. Similarly, intrathecal application of ER antagonists increased the spinal release of endomorphin-2 in female rats [63]. Although opioid receptors are expressed by TG and nodose ganglion neurons [26] the superficial laminae at the Vc/C_1–2_ region, the periacqueductal gray and rostral ventromedial medulla (RVM) also expresses a high level of opioid receptors [25], [58]. Selective ablation of mu opioid receptors in RVM greatly reduces stress-induced mechanical hyperalgesia [64]. This suggests that opioid receptor contributions to TMJ nociception likely involve peripheral as well as central sites of the nervous system. This interpretation is supported by our previous results [28]. Local application of morphine to the Vc/C_1–2_ reduced ATP-evoked TMJ unit activity similarly in both LE and HE rats. In contrast, systemic administration of morphine reduced activity only in LE rats and not in HE-treated rats. These findings suggest that the estrogen-dependent differences in opioid effects are not mediated locally within the Vc/C_1–2_, but rather involve higher-order mechanisms, such as descending control pathways.

One possible interpretation of these findings is that estrogen status is critical for regulating downstream effects on Vc/C_1–2_ neurons, such as the recruitment of descending control mechanisms from higher brain regions. Under low-estrogen conditions, vagal activity and endogenous opioids may more effectively engage rostral ventromedial medulla (RVM)-mediated inhibitory pathways. This interpretation is consistent with epidemiological studies indicating that postmenopausal women with lower estrogen status may display reduced TMD pain [27], [65]. Similarly, craniofacial pain, including TMD, has been reported to diminish after cessation of hormone contraceptive treatment in adult women [66]. Taken together, these observations suggest that interactions between hormonal status and autonomic systems play an important role in trigeminal pain modulation and may provide insight for developing new strategies for TMD management.

## Conclusion

In summary, vagotomy and naloxone selectively increased ATP-evoked activity of TMJ-responsive neurons under low estrogen conditions, but not under high estrogen conditions. These findings demonstrate that vagus nerve input and endogenous opioids act through estrogen-dependent common pathway to modify intra-TMJ responsive neurons at Vc/C_1–2_.

## Author contribution

A.T and D.A.B were responsible for the study concept and design. A.T and Y.M were responsible for data acquisition and analysis. A.T, Y.K and D.A.B were responsible for drafting major portions of the text and figures.

## CRediT authorship contribution statement

**Yuji Morimoto:** Validation, Resources. **Yuna Kani:** Visualization, Investigation, Data curation. **David A Bereiter:** Writing – review & editing, Validation, Conceptualization. **Akimasa Tashiro:** Writing – review & editing, Writing – original draft, Visualization, Validation, Supervision, Resources, Project administration, Methodology, Investigation, Funding acquisition, Formal analysis, Data curation, Conceptualization.

## Ethics approval and consent to participate

All experimental procedures were approved by the Committee of Research Facilities for Laboratory Animal Science at the National Defense Medical College, Japan, and were conducted in accordance with the NIH Guide for the Care and Use of Laboratory Animals (PHS Law 99–158; revised 2002).

## Declaration of Competing Interest

The authors declare that they have no known competing financial interests or personal relationships that could have appeared to influence the work reported in this paper

## Data Availability

The datasets generated and analyzed during the current study are available from the corresponding author upon reasonable request.
